# Documentation of Dementia as a Cause of Death Among Mexican-American Decedents With Dementia

**DOI:** 10.1093/geroni/igab046.228

**Published:** 2021-12-17

**Authors:** Brian Downer, Lin-Na Chou, Soham Al Snih, Cheyanne Barba, Yong-Fang Kuo, Mukaila Raji, Kyriakos Markides, Kenneth Ottenbacher

**Affiliations:** 1 University of Texas Medical Branch, Galveston, Texas, United States; 2 University of Alabama, Birmingham, Birmingham, Alabama, United States

## Abstract

There is lack of data on the frequency and correlates of dementia being documented as a cause of death in Hispanic populations. We investigated characteristics associated with dementia as a cause of death among Mexican-American decedents diagnosed with dementia. Data came from the Hispanic Established Populations for the Epidemiologic Study of the Elderly, Medicare claims files, and the National Death Index. Of the 744 decedents diagnosed with dementia before death, 26.9% had dementia documented as a cause of death. More health comorbidities (OR=0.38, 95% CI=0.25-0.57), older age at death (OR=1.05, 95% CI=1.01-1.08), and longer dementia duration (OR=1.09, 95% CI=1.03-1.16) were associated with dementia as a cause of death. In the last year of life, any ER admission with (OR=0.56, 95% CI=0.32-0.98) or without (OR=0.31, 95% CI=0.14-0.70) a hospitalization, more physician visits (OR=0.95, 95% CI=0.92-0.98) and seeing a medical specialist (OR=0.41, 95% CI=0.24-0.70) were associated with lower odds for dementia as a cause of death. In the last 30-days of life, any hospitalization with an ICU stay (OR=0.57, 95% CI=0.37-0.88) and ER admission with (OR=0.58, 95% CI=0.40-0.84) or without (OR=0.48, 95% CI=0.25-0.94) a hospitalization were associated with lower odds for dementia as a cause of death. Receiving hospice care in the last 30-days of life was associated with 2.09 (95% CI=1.38-3.16) higher odds for dementia as a cause of death. The possible under-documentation of dementia as a cause of death on death certificates may result in underestimation of healthcare resource need of dementia care for Mexican-Americans.

